# Radiotherapy as a metastasis directed therapy for liver oligometastases - comparative analysis between CT-guided interstitial HDR brachytherapy and two SBRT modalities performed on double-layer and single layer LINACs

**DOI:** 10.3389/fonc.2024.1478872

**Published:** 2024-11-04

**Authors:** Mateusz Bilski, Magdalena Peszyńska-Piorun, Katarzyna Konat-Bąska, Agnieszka Brzozowska, Katarzyna Korab, Ewa Wojtyna, Przemysław Janiak, Julia Ponikowska, Sylwia Sroka, Paweł Cisek, Jacek Fijuth, Łukasz Kuncman

**Affiliations:** ^1^ Department of Brachytherapy, Saint John’s Cancer Center, Lublin, Poland; ^2^ Department of Radiotherapy, Medical University of Lublin, Lublin, Poland; ^3^ Department of Radiotherapy, Saint John’s Cancer Center, Lublin, Poland; ^4^ Radiotherapy Planning Department, Copernicus Memorial Hospital in Lodz Comprehensive Cancer Center and Traumatology, Lodz, Poland; ^5^ Department of Brachytherapy, Lower Silesian Oncology, Pulmonology and Hematology Center, Wrocław, Poland; ^6^ Department of Mathematics and Statistics with e-Health Lab, Medical University of Lublin, Lublin, Poland; ^7^ Department of Medical Physics, Saint John’s Cancer Center, Lublin, Poland; ^8^ Department of Radiotherapy, Medical University of Lodz, Łódź, Poland; ^9^ Department of External Beam Radiotherapy, Copernicus Memorial Hospital in Lodz Comprehensive Cancer Center and Traumatology, Łódź, Poland

**Keywords:** brachytherapy, HDR, liver metastases, SBRT, SAbR, oligometastatic disease, CT-guided interstitial HDR brachytherapy, interventional radiotherapy

## Abstract

**Introduction:**

Surgical resection is gold standard for treatment of liver metastasis, locally ablative techniques including computer tomography (CT)-guided interstitial high-dose-rate (HDR) brachytherapy (CT-BRT) and stereotactic body radiotherapy (SBRT) have gained prominence as alternatives, offering comparable outcomes in selected patients. We aim to compare CT-BRT and SBRT - based on dosimetric analysis.

**Material and methods:**

Patients who underwent CT-BRT for oligometastatic, ≤4cm liver metastases between 2018 and 2024 were eligible. SBRT plans for Halcyon (SBRTh) and TrueBeam (SBRTtb) were prepared virtually. In the CT-BRT group CTV was equal to PTV, for SBRTh and SBRTtb planning, a 5 mm margin was applied to CTV to create PTV. Dose calculation was carried out with the TG-43 algorithm for CT-BRT and Anisotropic Analytical Algorithm for SBRTh and SBRTtb group. Descriptive statistics were used to compare the data. The Wilcoxon pairwise order test was utilized to compare dependent groups.

**Results:**

CT-BRT resulted in a more favorable dose distribution within PTVs for Dmean, D50, and D90, while SBRT showed better results for D98 and V27.5Gy. No significant differences were observed for V25Gy between CT-BRT and SBRTtb, but SBRTh favored over CT-BRT. For OARs, CT-BRT plans showed better values for V5, V10, and V11.6Gy in the uninvolved liver volume. There were no significant differences in dose distribution for the duodenum, bowel, and heart. SBRT modalities performed better in the kidney. CT-BRT had improved dose distribution in the esophagus, great vessels, ribs, skin, spinal cord, and stomach compared to SBRT.

**Conclusions:**

CT-BRT could be a viable alternative to SBRT for certain patients with liver malignancies.

## Introduction

The concept of oligometastatic disease (OMD) was first proposed by Hellman and Weichselbaum in 1995 and was later classified by The European Society for Radiotherapy and Oncology and the European Organization for Research and Treatment of Cancer (1–3). It defines a state of limited metastatic spread characterized by a restricted number of lesions (usually defined as up to 5) in one or a few organs. This idea redefined the management approach, emphasizing the potential for aggressive radical local therapies to achieve better disease control and improve patient outcomes. Liver metastases represent a common manifestation of advanced cancer, originating from primary tumors such as gastrointestinal, breast, prostate cancer, melanomas, neuroendocrine tumors, and sarcomas (4). The management of liver metastases includes a variety of treatment options, such as surgical resection, ablative therapies, and systemic therapies. While surgical resection has been considered the gold standard for resectable lesions, non-surgical locally ablative techniques, such as radiofrequency ablation (RFA), microwave ablation (MWA), precision radiotherapy (SBRT, BRT), and embolization techniques (chemoembolization or radioembolization) have gained prominence as alternative therapeutic modalities, offering comparable outcomes in selected group of patients (5–8). CT-guided interstitial HDR brachytherapy (CT-BRT), also referred to as interventional radiotherapy, employs CT-based real-time imaging to precisely deliver high doses of radiation directly to the tumor, making it a promising option for liver oligometastases. This ablative method involves inserting a radioactive source, such as Iridium-192, into tumor lesions through catheters placed under CT guidance. Transarterial radioembolization (TARE) is another brachytherapy technique that delivers radioactive microspheres directly into the tumor’s vasculature via transarterial infusion (9). Unlike RFA and MWA, CT-BRT overcomes limitations related to tumor size and location, including proximity to critical structures like the liver hilum or major blood vessels (10). Similarly, SBRT, which delivers highly conformal and ablative doses of radiation in a limited number of fractions, has demonstrated excellent tumor control while minimizing radiation-induced toxicity. There is growing support for the use of SBRT in liver data from both retrospective and prospective studies (11–15). Despite the increasing use of radiation therapy for the treatment of liver metastases, comprehensive dosimetric analyses comparing different treatment modalities rem ain rare (16–19). This study aims to fill this gap by providing a detailed dosimetric analysis of HDR CT-BRT and SBRT performed using Halcyon and TrueBeam in the context of local treatment of oligometastatic liver lesions.

## Materials and methods

The study analyzed 30 patients with oligometastatic liver disease from various primary cancers. All patients underwent HDR CT-BRT. SBRT plans for Halcyon (SBRTh) and TrueBeam (SBRTtb) were prepared. The patients were treated at XXX between 2018 and 2024. Metastases were located in different liver segments, allowing for a comparison of doses between different organs at risk (OARs). Metastases with an upper diameter of 4 cm were included. A single fraction of 25 Gy was used in the CT-BRT and both SBRT modalities. The study was conducted in accordance with the Declaration of Helsinki and was approved by the local Ethics Committee.

### Common planning rules for all modalities

In the case of CT-BRT clinical target volumes (CTV), no additional margin was added because the applicators are positioned directly within the metastases and move with them as the patient breathes (CT-BRT CTV was equal planning target volume (PTV)). However, for SBRTh and SBRTtb planning, a 5 mm margin was applied to CTV to create PTV due to uncertainties related to liver movement during the procedure. **Table 1** (v) presents the dose constraints to OARs (20–23).

**Table 1:** Dose constraints applied for the comparative analysis.

OAR	Dose (D) or volume (V) constrains
Uninvolved Liver	V11.6Gy<700cc V10Gy<700cc V5Gy<66% D66%<10Gy
Ribs	Dmax< 30Gy D1cc< 23Gy
Bowel	Dmax<15.4Gy D5cc<11.9Gy
Kidney (Right)	D1cc <18Gy Dmean<6Gy D100cc<9.5Gy
Heart	Dmax<22Gy
Skin	Dmax<26Gy D10cc<23Gy
Stomach	Dmax <12.4Gy D10cc<11.2 Gy D1cc <15Gy
Duodenum	Dmax<12.4Gy D5cc<11.2Gy D10cc<9Gy D1cc<15Gy
Gallbladder	Dmax<20Gy
Biliary tract	Dmax<25Gy
Spinal Cord	Dmax <14Gy D0.35cc<10Gy D1.2 cc<7Gy D1cc<14Gy
Great Vessels	D1cc< 27Gy
Esophagus	Dmax<24Gy D1cc<15Gy

### CT-BRT procedure

In our previous publications, we have described the rules of application for CT-BRT (19, 24, 25). All selected patients underwent general anesthesia. After contrast administration, the simulation began with the radiation oncologist (RO) using CT to match the upper and lower borders of the tumor. These borders were then drawn on the patients’ skin before the application started. The RO used 200 or 320 mm long applicators (Varian, Inc.) for direct application, while a 32-slice CT scanner with real-time fluoroscopic imaging was used during the procedure. Following the manual part of the procedure, a CT scan with 1.5-3 mm slice thickness was performed. The RO delineated CTVs and OARs using fusion with diagnostic CT and/or magnetic resonance imaging (MRI) and/or positron emission tomography (PET/CT). The source step was set to 5 mm, and in most cases, dose volume optimization was performed using inverse planning as a starting point for manual optimization. All patients were planned in the BrachyVision planning system version 10 or 16 (Varian Medical Systems, Inc.), and dose calculation was carried out with the TG-43 algorithm. Treatment plans were delivered using BRAVOS or GammaMed iX HDR iridium 192 after loaders (Palo Alto, USA). The treatment time was defined by the radioactive source activity, with a nominal source value of 10 Ci applied for this analysis. Application and planning times were not evaluated due to the dependency on several factors, including the experience of the RO and medical physicist, the number of applicators used, and the complexity of the plan.

### Virtual SBRT treatment planning for Halcyon and TrueBeam

The same CT datasets were utilized for simulating teleradiotherapy and brachytherapy planning. Planning included the creation of plans for the Varian Truebeam HD linear accelerator and Halcyon linear accelerator by Varian. Within the Varian Eclipse Treatment Planning System (version 16.01), plans were formulated based on the VMAT (Volumetric Modulated Arc Therapy) technique. Each plan incorporated two VMAT arcs with a photon energy of 6 MV, all calculated using the Anisotropic Analytical Algorithm (AAA). For a standard teleradiotherapy treatment plan using 4DCT, a 5 mm margin in each direction was added to the CTV as part of the Internal Target Volume. Additionally, CT scans prepared for brachytherapy planning were utilized to prepare hypothetical teleradiotherapeutic plans, defining the PTV as CTV + 5 mm margin. The estimation of irradiation time was facilitated by a customized program, necessary due to the unavailability of this function within the Varian treatment planning system (TPS). Utilizing information obtained from the TPS regarding the value of the gantry angle and the speed of gantry rotation between subsequent control points, the total estimated irradiation time on the device was calculated.

### Explanation of selected conformity indices

Two indices: R50% and Paddick Index (PCI) were selected for the purpose of this analysis (26).

Modified Gradient Index (R50%)


$$\text{R}50\%=\frac{Vol(50\%)}{PTV\;V100\%}$$

where

• Vol (50%): the volume of the patient covered by half of the prescription isodose,

• PTV V100% - volume of the target covered by prescription isodose.

Paddick Index (PCI)


$$\text{PCI}=\frac{{\left(PTV\;V100\%o\;r\;CTV\;V100\%\right)}^{2}}{\left(V\;PTV\;or\;V\;CTV)*Vol(100\%\right)}$$

where:

• PTV V100% - volume of the target covered by prescription isodose,

• CTV V100% - volume of the target covered by prescription isodose,

• V PTV-PTV volume,

• V CTV-CTV volume,

• Vol (100%): the volume of the patient covered by prescription isodose.

### Statistical analysis

The measurable parameters were presented using median, standard deviation, and ranges. The Shapiro-Wilk W test was used for normality check. The Wilcoxon pairwise order test was utilized to compare dependent groups, with p<0.05 regarded as statistically significant. STATISTICA 13.0 software by StatSoft, Poland was used for calcultations.

## Results

The median liver volume was 1398.63 cm^3 (range 1005.14-2209.85). The median size of the metastatic lesion was 3.15 cm (range 1.9-4.0). The median clinical target volume was 9.26 cm^3 (range 2.91-29.71). The median planning target volume (PTV) differed for CT-BRT and SBRT, being 9.26 and 25.42, respectively (**Table 2**).

**Table 2:** Basic characteristics of the assessed parameters.

Parameters	Median (range)
Lesion size (cm)	3.15 (1.90-4.00)
CTV(cm^3^)	9.26 (2.91-29.71)
PTV(cm^3^)	
I-RT	9.26 (2.91-29.71)
SBRT_tb_	25.42 (11.02-59.16)
SBRT_h_	25.42 (11.02-59.16)
Liver volume (cm^3^)	1398.63 (1005.14-2209.85)
Treatment time (min)	
I-RT	8.11 (2.95-13.17)
SBRTtb	4.17 (2.79-6.11)
SBRTh	7.14 (6.13-9.54)

### PTVs dose coverage: a crucial analysis

**Figure 1:**
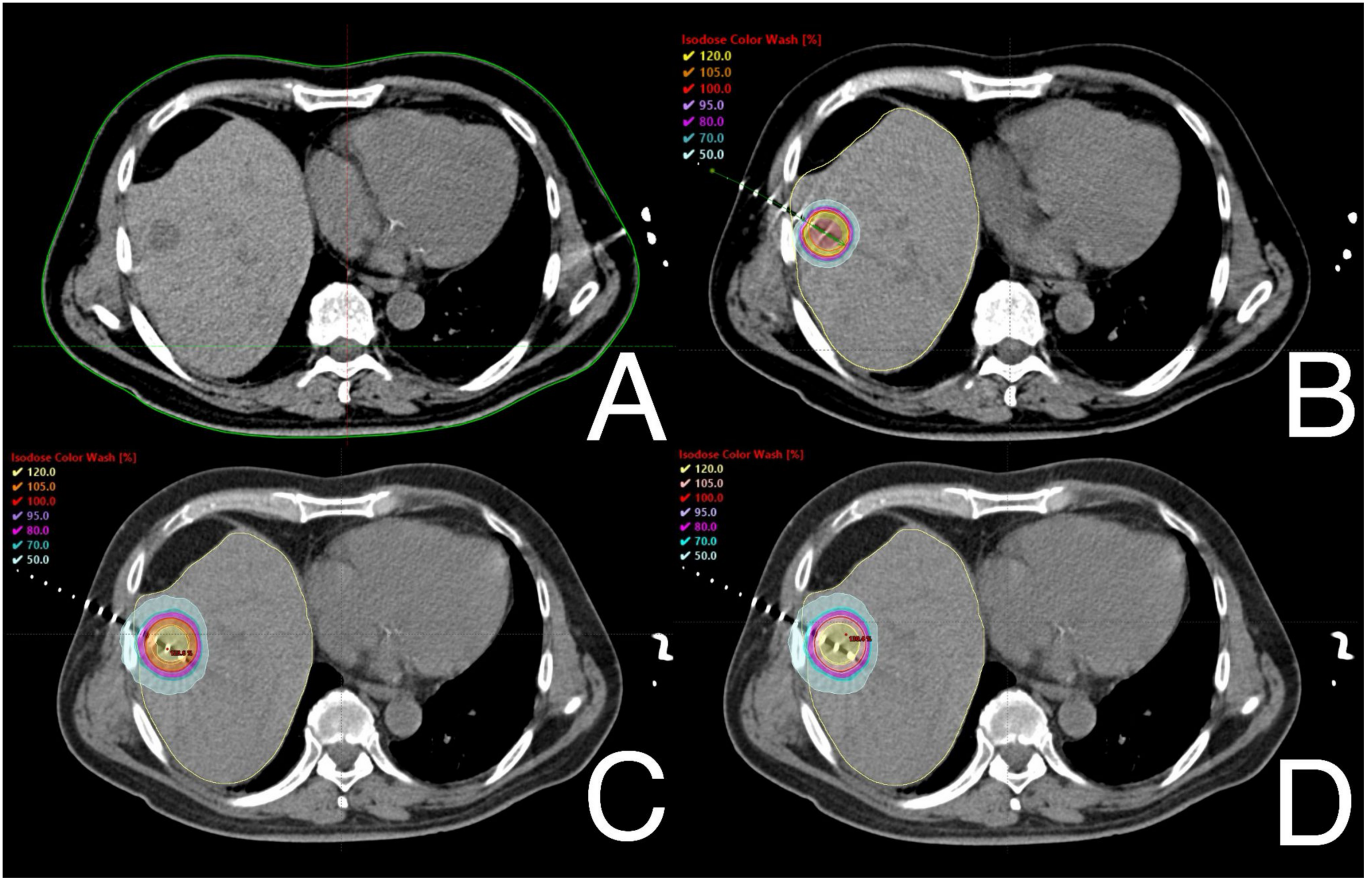
Example of dose distribution profiles within PTVs of one of selected patients with metastasis in fifth liver segment: diagnostic scan taken before treatment **(A)**, CT-BRT **(B)**, SBRTtb **(C)** and SBRTh **(D)**.

The statistical analysis revealed significant differences in certain dosimetric variables for PTVs. Plans developed for CT-BRT showed significantly better median D50, D90, and Dmean values. However, there were no differences in the Dmean and D50 parameters between both SBRT modalities. SBRT plans also demonstrated superior results in terms of D98 and V23.75Gy compared to those prepared for CT-BRT. There were no significant differences related to V25Gy between SBRTtb and CT-BRT. On the other hand, SBRTtb plans exhibited better D98, V23.75Gy, and V25 values compared to those prepared for SBRTh and CT-BRT. **Figure 1** illustrates the dose distribution within PTVs for a selected liver metastasis. A detailed overview of the dose distribution within PTVs across the selected techniques is provided in **Supplementary Material Table S1**.

### Comparison of selected indices

**Figure 2:**
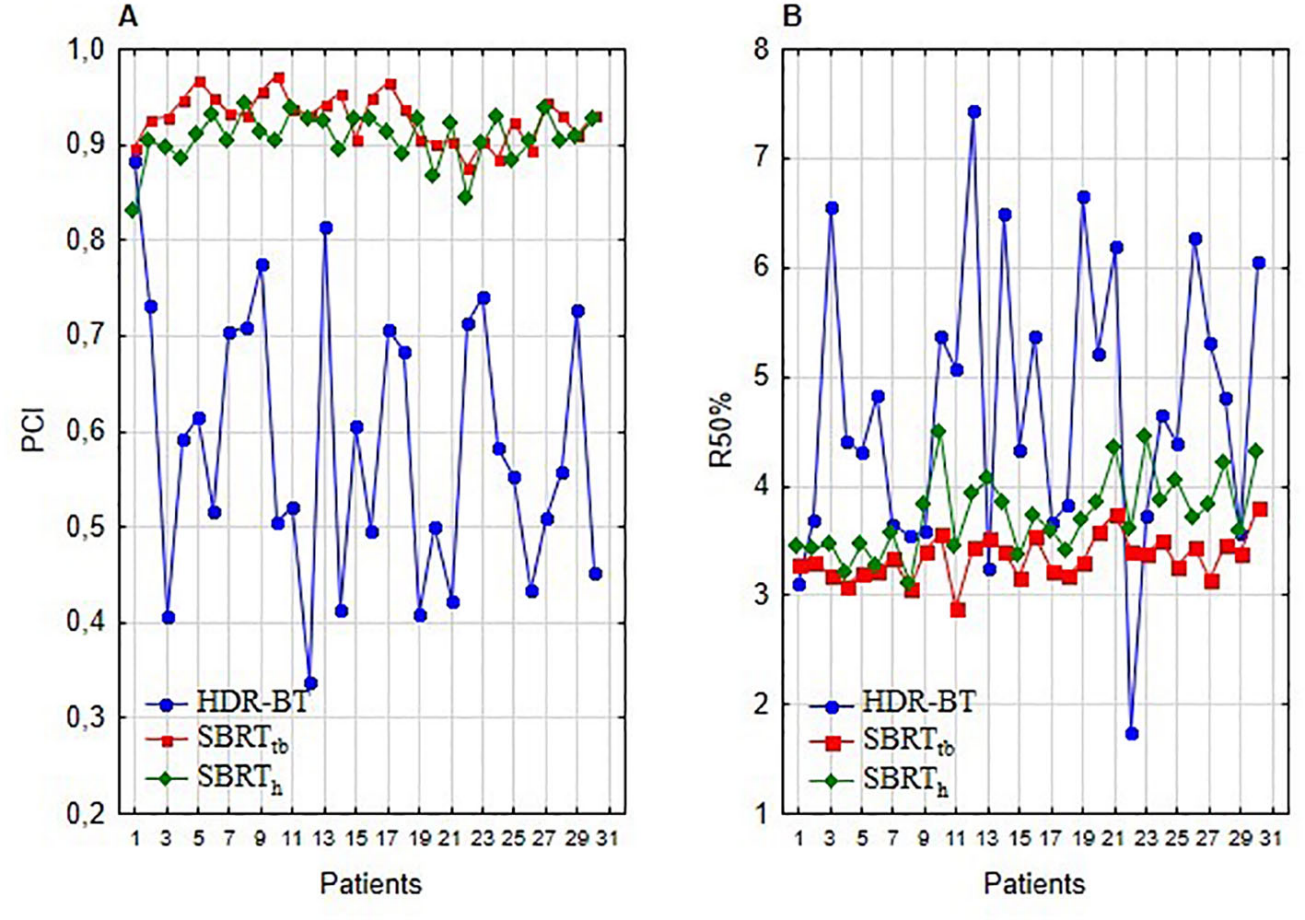
Plan comparison of HDR-BRT, SBRTtb, and SBRTh: **(A)** illustrates PCI index values, and **(B)** presents R50% index values.

In comparing techniques, individual indices revealed a notable difference. The best median PCI was attained with SBRTtb plans, outperforming both CT-BRT (p<0.001) and SBRTh (p<0.001). This trend was also noticed for the R50% value (**Table 3**, **Figure 2**). The results strongly indicate SBRTtb as the preferred option when assessing selected indices.

**Table 3:** The comparison of index values between I-RT, SBRTtb, and SBRTh modalities.

Index	I-RT	SBRT_tb_	SBRT_h_	Analysis
**PCI**	0.57±0.14	0.93±0.02	0.91±0.03	I-RT-SBRTtb*: p<0.0001 I-RT-SBRTh*: p<0.0001 SBRTtb*-SBRTh: p=0.001
**R50%**	4.54±1.30	3.37±0.3	3.70±0.37	I-RT-SBRTtb*: p<0.0001 I-RT-SBRTh*: p=0.0004 SBRTtb*-SBRTh: p<0.0001

### Results for organs at risk

#### Uninvolved liver volume

**Figure 3:**
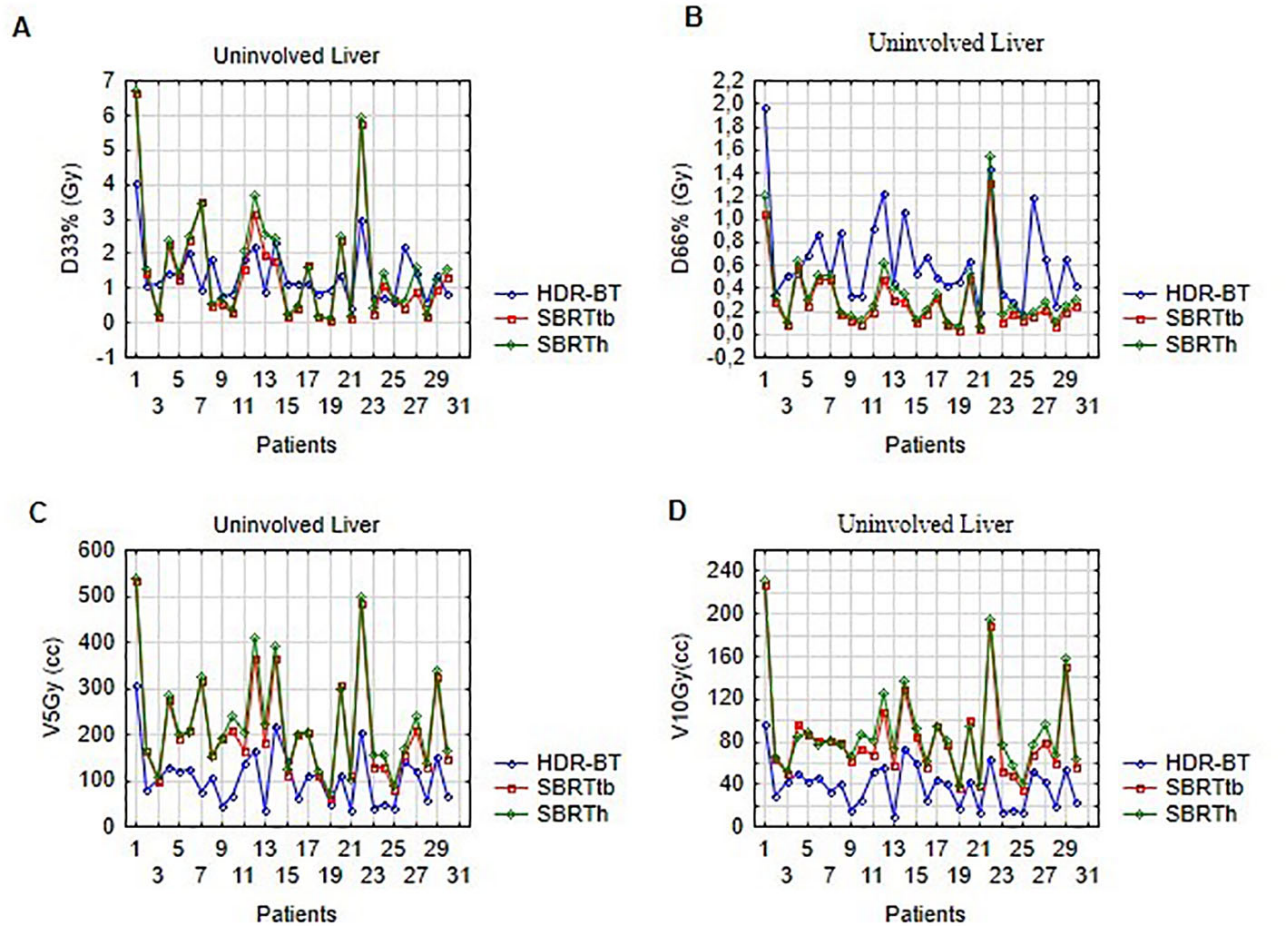
Dose distribution in uninvolved liver volume for selected ablative techniques: **(A)** illustrates D33% (Gy), **(B)** shows D66% (Gy), **(C)** presents V5Gy (cc), and **(D)** displays V10Gy (cc) for HDR-BT, SBRTtb, and SBRTh.

The lowest values for V5Gy, V10Gy, and V11.6Gy for the uninvolved liver were obtained with CT-BRT plans (p<0.001). SBRTtb achieved better results than SBRTh for the same parameters. However, for D66%, the dose analysis showed lower values for both SBRT techniques. Plans prepared for SBRTtb presented better results than for SBRTh for all analyzed liver variables. No significant differences were observed for D33% when comparing CT-BRT and both SBRT modalities (p=0.34 and p=0.88, respectively) (**Figure 3**; **Supplementary Table S2**).

#### Dose distribution in selected OARs favoring CT-BRT plans

**Figure 4:**
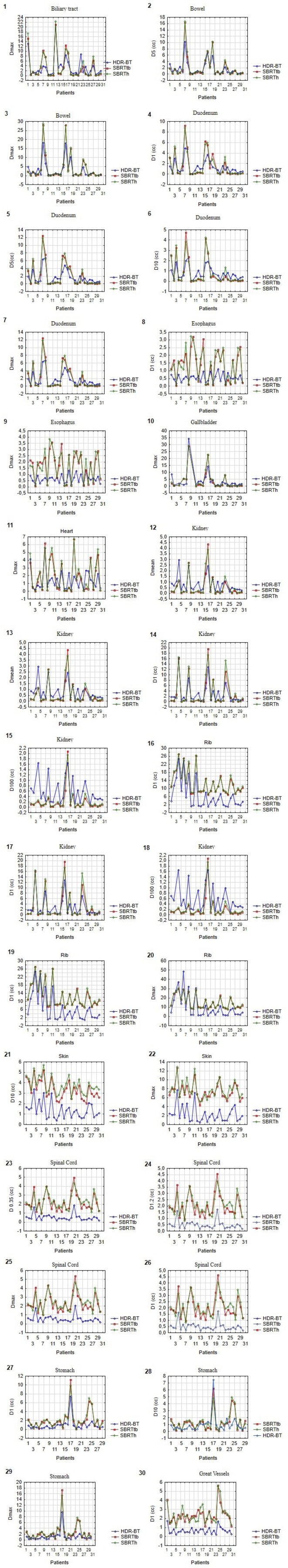
Dose distribution in OARs between selected ablative radiotherapy modalities.

CT-BRT plans achieved better results for the esophagus (Dmax, D1cc) and great vessels (D1cc) compared to plans prepared for SBRTtb and SBRTh. Similar results were obtained for the spinal cord and skin for all analyzed parameters (p<0.001 for all). CT-BRT plans also achieved the lowest dose levels for ribs (Dmax, D1cc) and stomach (Dmax, D1cc, D10cc) (**Figure 4**; **Supplementary Table S2**).

#### Dose distribution in selected OARs favoring SBRT performed on TrueBeam

Regarding the duodenum, statistically significant differences favoring SBRTtb over SBRTh were observed for Dmax, D1cc, and D10cc (p<0.01 for all). However, no significant differences in Dmax, D1cc, D5cc, or D10cc were found between CT-BRT and stereotactic techniques. Notably, SBRTtb plans demonstrated lower median values for bowel: Dmax, D5cc; biliary tract: Dmax; gallbladder: Dmax; kidney R: D1cc; and heart: Dmax, as compared to SBRTh plans. Similar organs displayed no differences compared to CT-BRT plans. Furthermore, SBRTtb plans yielded superior outcomes for stomach: D10cc compared to SBRTh (p=0.03). Regarding kidney R: Dmean and D100cc, differences were observed between SBRTtb and CT-BRT as well as SBRTh, with SBRTtb plans presenting the lowest median values for these parameters (**Figure 4**; **Supplementary Table S2**).

#### Dose distribution in selected OARs for SBRT Halcyon

The analysis of dose distributed within OARs, including the duodenum, bowel, biliary tract, gallbladder, esophagus, kidney R (only D1cc), heart, great vessels, ribs, skin, spinal cord, and stomach, indicates that the SBRTh method is not the preferred option when compared to CT-BRT and SBRTtb plans. It is worth noting that SBRTh plans exhibited better results only for kidney R: Dmean and D100cc, in comparison to CT-BRT plans (**Figure 4**; **Supplementary Table S2**).

## Discussion

We compared radiation doses in target volumes and OARs in 30 patients with liver metastases up to 4 cm across all liver segments, evaluating CT-BRT and virtual SBRT plans for Halcyon and TrueBeam systems. To the best of our knowledge, this is the fourth comparative dosimetric analysis of hepatic malignancies treated with HDR brachytherapy (BT) vs. SBRT (16–18), and the second in which the recommended doses from BT and SBRT have been standardized in terms of applied dose and number of fractions (19). A recently published analysis compared the treatment of liver metastases with intrathecal HDR-BTvs SBRT performed with CyberKnife and Elekta Versa HD LINAC (19). All above studies indicate the benefit of BT in terms of: more favorable dose distribution within target volume and dose reduction in healthy liver volume. Nevertheless, the studies also underscore the need for further research to determine the ideal application for each treatment modality (16–19).

Historically, local treatment of liver metastases was palliative. The concept of OMD has increased indications for local treatments, including brachytherapy. OMD now includes patients with primary synchronous or metachronous limited metastases and those with prior polymetastatic disease achieving induced OMD or oligoprogressive/oligopersistent status after systemic therapy. The role of ablative treatment in this context is to help maintain patients on their current line of systemic therapy (25). Local treatment of metastases in OMD requires high doses: Biological Equivalent Dose-BED>100Gy, BED10 and protection of OARs due to the reduction of toxicity of radiotherapy treatment along with systemic treatment or immunotherapy (14). Therefore, CT-BRT, due to its dose distribution, seems to be a method worth considering for the treatment of liver metastases. Recently, the European Society of Medical Oncology (ESMO) includes liver HDR-BT as a treatment option in its clinical treatment guidelines for HCC and metastatic colorectal cancer (8). Another indication where it is particularly important to protect the surrounding OARs with the simultaneous need for a high dose of radiation in the target volume is the growing group of patients requiring re-irradiation. This situation particularly applies to re-irradiation cases type 1 and 2 according to Andratschke where there is high concern of toxicity from cumulative doses to healthy tissues (27). When comparing dose distribution between CT-BRT and SBRT, usage of different dosimetry calculation (TG-43 for CT-BRT and AAA for SBRT) should be discussed. The TG-43 algorithm does not take into account tissue heterogeneity, attenuation and scattering in the applicator, and the effects of the patient boundary, unlike the SBRT dose distribution calculation algorithm (28, 29). Several studies suggest that in homogeneous tissue with plastic applicators and no nearby air spaces, the impact of algorithms on dosimetric results is neglectable (29). In our study, which focused on OARs near the liver (with a density of 1.06 g/cm³, similar to water), these conditions were fully met.

Liver SBRT is commonly administered in a fractionated manner, typically involving 3 to 8 fractions of 6-20 Gy (14, 30). In our investigation, we prescribed a single fraction of 25 Gy for virtual SBRT planning, which aligns with the prevalent practice of utilizing high-dose single fractions for liver metastases treatment with CT-BRT. Notably, previous reports have also documented the use of single fraction SBRT. For instance, studies by Laliscia et al. and Herfarth et al. have explored the effectiveness of single fraction of 22-24 Gy for liver metastases from gynecological/colorectal (31, 32). Furthermore, retrospective and prospective trials have made it evident that higher BED10 levels surpassing 150 Gy can significantly enhance local control rates (LCR) for liver metastases (11, 12, 15, 33). The advancement of modern radiotherapy techniques has facilitated the precise delivery of higher doses to target volumes while safeguarding organs at risk, thereby rekindling interest in SBRT trials utilizing single high doses for liver metastases. Notably, a phase 1 dose-escalation trial demonstrated promising outcomes with the prescription of 35-40 Gy, resulting in a four-year LCR of 96.6% and minimal only G2 toxicity of 9% (13). These findings suggest that the application of single-fraction SBRT for liver metastases can be carried out safely. Nevertheless, further confirmation through future phase III trials is warranted. It is pertinent to note that dosimetric analyses involving single-fraction SBRT, such as the one conducted within our study, can offer valuable insights to inform the design of future trials. It is well known that during interstitial brachytherapy procedure extreme maximal doses are generated around the needle, even above 1000 Gy. It can be translated for comparison into isocenter doses while SBRT planning. From the perspective of SBRT trials the importance of high isocenter doses was already proven in primary and secondary lung cancers (34, 35). The predictive value of maximum isocenter BED was confirmed in the German Society for Radiation Oncology (DEGRO) analysis where SBRT was used for liver oligometastases (36). In univariate (HR (CI) 0.993 (0.989-0.997), p<0.001) and multivariate (HR (CI) 0.99(0.98-1.0), p=0.002) analysis isocenter BED10 doses, above 150 Gy were predictive for better LCR of >80% at 2 years (36). These observations potentially indicate a clear benefit from the use of brachytherapy in the treatment of liver metastases. However, this requires confirmation in future studies. Another point worth considering is that the mean dose in target volume might also have a predictive value. It was already proven that the mean dose in target volume is a prognostic factor in regard to LCR, the mean BED10 doses lower than 130 Gy led to higher local recurrence rates (37). We have evaluated Dmean dose in single fractions for CT-BRT and SBRT plans and this is the first report of this kind in the literature. For CT-BRT Dmean dose was 61.13 ± 10.64 and it was more than two times higher than for virtual SBRT plans, 28.58 ± 0.32 for SBRTtb and 28.53 ± 0.29 for SBRTh. This may explain why generally lower fraction doses can be prescribed during the CT-BRT procedure in comparison to SBRT planning enabling achieving similar rates of LCR (17). So it has to be taken into consideration that the best predictive factors for LCR in liver metastases SBRT or CT-BRT might be high isocenter BED10 doses, maximal doses, and mean doses within target volumes rather than minimal doses (D98). Assuming that CT-BRT might be a great alternative to EBRT-based modalities.

In our examination, the dose constraints for the unaffected liver were satisfied across all plans for the selected treatment modalities. Given the escalating frequency of reirradiation for various oligometastatic clinical scenarios, such as oligoprogressive, oligopersistent, and oligorecurent disease, the minimization of radiation exposure to healthy liver tissue assumes particular significance. This is essential to preserve optimal liver function during long-term systemic treatment, which may also have implications for its functioning. As per the dose constraints, the CT-BRT demonstrated the lowest dose distribution consistent with V5, V10, and V11.6 Gy values.

Treatment of peripherally localized liver metastases can be challenging. Proximity to the ribs and skin can make it very difficult to administer a sufficiently high dose to provide local control while trying to limit the risk of complications such as fracture, chronic pain, intercostal nerve damage, or skin ulceration. It was reported that the probability of rib fracture, after SBRT for lung tumors, after 4 years of follow-up, was 47.7% and 12.9% (p = 0.0184) when taking into account threshold Dmax of 54 Gy. However, in this analysis, no patients complained about pain (38). In a more recent analysis of 243 patients after liver tumors SBRT, 6.2% of patients developed rib fractures after a median of 7 months from the procedure. More than 50% of patients presented additional chest wall pain (39). On univariate analysis, V30, V40, D30cm3, and Dmax were independent predictive factors for rib fracture development (39). In the pooled analysis of 57 studies, involving 5985 patients, chest wall pain and rib fracture after SBRT were present in 11% (95%CI, 8.0-14.4) and 6.3% (95%CI,3.7-9.7), respectively (40). This study showed also that a distance smaller than 1.6-2.5 cm from the chest wall and chest wall or rib volumes receiving more than 30 Gy were strong predictors of fracture and pain (40). In our cohort, all of the modalities met dose constraints with the best results presented by CT-BRT plans. In SBRT, respiratory motion is crucial due to high doses and steep gradients. Liver movement from respiration can reach several centimeters, causing suboptimal tumor dosing and excess normal tissue irradiation. Various respiratory control methods available in liver SBRT include voluntary breath hold, abdominal compression, free breathing gating, and free breathing (41). Nevertheless, to ensure adequate target coverage, additional margins are frequently utilized to counteract the adverse effects of intra- and inter-fractional organ movements, thereby increasing the volume of normal tissues exposed to radiation (42). ​​The variability in the position of liver tumors significantly depends on their location within the liver segments. Tumors situated in peripheral segments exhibit increased intra-fraction movement compared to those located centrally. For instance, tumors in peripheral segments tend to experience greater positional variability due to these regions’ anatomical and physiological dynamics.

Different mobility and margins are reported depending on the respiratory motion compensation technique used (43). Based on the results of a recently published systematic review intra-fraction variability for free breathing can be even 4.2 mm, 5.4 mm and 9.7 mm in left-right (LR), anterior-posterior (AP) and superior-inferior (SI) directions respectively (43). For voluntary breath hold, abdominal compression techniques laser margins are required with inter- and intra-fraction variability not exceeding 3 mm (43). The authors concluded that for breath-hold treatments, a symmetrical weighted-mean planning target volume (PTV) margin of 6 mm is appropriate (43). In contrast, for free breathing and abdominal compression, asymmetric weighted-mean PTV margins of 4 mm in the AP direction and 6 mm in the SI and LR directions are recommended (43).

CT-guided interstitial HDR brachytherapy is highly effective but requires specialized expertise and infrastructure. In contrast, SBRT is non-invasive, easier to implement, and more feasible for routine use in many centers. Moreover, there is a lack of clear guidelines regarding the specific requirements for CT-BRT use, which contrasts with procedures like TARE, where well-established protocols and criteria guide clinical practice (9). CT-BRT dose distribution depends heavily on needle placement, and suboptimal implantation cannot be fully corrected through planning, highlighting the need for procedural precision (44). As a result, achieving optimal dosimetric constraints may be more difficult in HDR brachytherapy compared to SBRT, where treatment plans are more standardized and reproducible.

Liver brachytherapy is a minimally invasive procedure. In our analysis the application phase can last 15-25 minutes using the described technique. This procedure can be performed under local or general anesthesia, and patients with contraindications for general anesthesia can still undergo CT-BRT. It is pertinent to note that when planning SBRT, many centers utilize fiducial markers, which are placed inside the liver parenchyma or directly in the tumor, thereby rendering SBRT a minimally invasive technique. Possible marker migration, reported in the literature and occurring in up to 8% of patients, should also be taken into account (45). With the single fraction procedure, the hospital stay is typically short, approximately 2-3 days, prompting many patients to consider CT-BRT when there is an alternative.

Our analysis has inherent limitations. Firstly, it is a retrospective study. Additionally, the study compared patients who were eligible for CT-BRT and created alternative treatment plans for these cases, which introduces a selection bias. These biases arise from the fact that the eligibility criteria for lesions qualifying for CT-guided brachytherapy (CT-BRT) are narrower than for SBRT. Another bias arises from the fact that physicists retrospectively preparing SBRT plans had more time for planning. In contrast, brachytherapy was performed directly with unmodified treatment plans, and medical physicists had limited time for CT-BRT planning. Moreover, it is noteworthy that we utilized a 5 mm PTV margin for SBRT planning, which is smaller than the margin typically employed in many centers. This smaller margin provides an additional advantage for SBRT modalities during dosimetric comparison. In our opinion, the selection of the radiotherapy modality should always be deliberated with patients and within multidisciplinary tumor boards. Dosimetric comparison studies may aid in determining the optimal, case-based qualification for liver MDT among the various radiotherapy techniques available.

## Conclusions

CT-BRT achieved a more favorable dose distribution within PTVs based on Dmean, D50, and D90, with D98 and V27.5Gy being better for both SBRT modalities. For OARs, CT-BRT showed better values for V5, V10, and V11.6Gy in uninvolved liver volume, esophagus, great vessels, ribs, skin, spinal cord, and stomach compared to SBRT. SBRT modalities had better outcomes in the kidney. These findings suggest CT-BRT as a viable alternative to SBRT for selected liver malignancy patients.

## Data Availability

The raw data supporting the conclusions of this article will be made available by the authors, without undue reservation.
