# Supplementary material for: Radiotherapy as a metastasis directed therapy for liver oligometastases - comparative analysis between CT-guided interstitial HDR brachytherapy and two SBRT modalities performed on double-layer and single layer LINACs
Source: Front Oncol. 2024 Nov 4;14:1478872. doi: 10.3389/fonc.2024.1478872 (PMC11570579; doi:10.3389/fonc.2024.1478872)
Supplement: Supplementary file 1 [file DataSheet1.docx]

Suplementary materials:

Table S1. The comparison of doses distributed to PTVs between I- RT, SBRTtb and SBRTh.

| **Variable** | **I -RT** | **SBRT_tb_** | **SBRT_h_** | **Analysis** |
| --- | --- | --- | --- | --- |
| **Dmean (Gy)** | 61.13±10.64 | 28.58±0.32 | 28.53±0.29 | I-RT* - SBRTtb: p<0.0001  I-RT* - SBRTh: p<0.0001  SBRTtb - SBRTh: p=0.14 |
| **D50% (Gy) (median)** | 49.66±6.44 | 28.71±0.60 | 28.66±0.54 | I-RT* - SBRTtb: p<0.0001  I-RT* - SBRTh: p<0.0001  SBRTtb - SBRTh: p=0.78 |
| **D90% (Gy)** | 28.12±1.49 | 25.55±0.42 | 25.26±0.45 | I-RT* - SBRTtb: p<0.0001  I-RT* - SBRTh: p<0.0001  SBRTtb* - SBRTh: p=0.004 |
| **D98% (Gy)** | 22.61±1.60 | 24.30±0.63 | 23.92±0.79 | I-RT - SBRTtb*: p=0.0001  I-RT - SBRTh*: p=0.0005  SBRTtb* - SBRTh: p=0.02 |
| **V23.75Gy (%)** | 96.95±1.85 | 99.31±1.17 | 98.45±2.15 | I-RT - SBRTtb*: p=0.0001  I-RT - SBRTh*: p=0.002  SBRTtb* - SBRTh: p=0.004 |
| **V25Gy (%)** | 95.32±2.08 | 94.43±3.15 | 92.08±3.87 | I-RT - SBRTtb: p=0.26  I-RT - SBRTh*: p=0.0005  SBRTtb* - SBRTh: p=0.03 |

Table S2. Dose distribution values in OARs between selected radiotherapy modalities.

| **OARs** | | **I-RT** | **SBRT_tb_** | **SBRT_h_** | **Analysis** |
| --- | --- | --- | --- | --- | --- |
| **Organ** | **DVH value** | **median±SD** | **median±SD** | **median±SD** |  |
| **Uninvolved Liver** | V5Gy(%) | 7.16±4.73 | 12.90±8.53 | 14.30±8.59 | I-RT* - SBRTtb: p<0.001  I-RT* - SBRTh: p<0.001  SBRTtb* - SBRTh: p<0.001 |
|  | V5Gy (cm3) | 108.81±61.99 | 187.63±114.77 | 199.04±116.63 | I-RT* - SBRTtb: p<0.001  I-RT* - SBRTh: p<0.001  SBRTtb* - SBRTh: p<0.001 |
|  | V9.1Gy | 48.37±23.90 | 86.17±52.48 | 90.77±53.30 | I-RT* - SBRTtb: p<0.0001  I-RT* - SBRTh: p<0.0001  SBRTtb* - SBRTh: p=0.001 |
|  | V10Gy | 41.35±20.28 | 75.12±43.01 | 79.03±43.17 | I-RT* - SBRTtb: p<0.0001  I-RT* - SBRTh: p<0.0001  SBRTtb* - SBRTh: p=0.0004 |
|  | V11.6Gy (cm3) | 32.07±15.44 | 58.08±31.02 | 61.63±30.18 | I-RT* - SBRTtb: p<0.001  I-RT* - SBRTh: p<0.001  SBRTtb* - SBRTh: p=0.0003 |
|  | D33%(Gy) | 1.16±0.78 | 1.00±1.59 | 1.465±1.62 | I-RT - SBRTtb: p=0.88  I-RT - SBRTh: p=0.34  SBRTtb* - SBRTh: p<0.001 |
|  | D66% | 0.52±0.40 | 0.19±0.29 | 0.25±0.33 | I-RT - SBRTtb*: p<0.0001  I-RT - SBRTh*: p<0.0001  SBRTtb* - SBRTh: p<0.0001 |
| **Duodenum** | Dmax | 1.00±1.79 | 0.25±3.20 | 0.29±3.10 | I-RT - SBRTtb: p=0.85  I-RT - SBRTh: p=0.96  SBRTtb* - SBRTh: p=0.01 |
|  | D5cm³ | 0.68±0.86 | 0.16±1.78 | 0.19±1.66 | I-RT - SBRTtb: p=0.66  I-RT - SBRTh: p=0.60  SBRTtb - SBRTh: p=0.06 |
|  | D10cm³ | 0.58±0.63 | 0.13±1.36 | 0.15±1.28 | I-RT - SBRTtb: p=0.14  I-RT - SBRTh: p=0.26  SBRTtb* - SBRTh: p=0.003 |
|  | D1cm³ | 0.84±1.31 | 0.21±2.44 | 0.25±2.34 | I-RT - SBRTtb: p=0.79  I-RT - SBRTh: p=0.77  SBRTtb* - SBRTh: p=0.01 |
| **Bowel** | Dmax | 1.06±4.69 | 0.70±7.46 | 0.92±7.56 | I-RT - SBRTtb: p=0.75  I-RT - SBRTh: p=0.99  SBRTtb* - SBRTh: p=0.002 |
|  | D5cm³ | 0.86±2.29 | 0.51±3.62 | 0.64±3.73 | I-RT - SBRTtb: p=0.68  I-RT - SBRTh: p=0.93  SBRTtb* - SBRTh: p<0.0001 |
| **Biliary tract** | Dmax | 1.72±4.40 | 0.65±5.30 | 0.85±5.56 | I-RT - SBRTtb: p=0.57  I-RT - SBRTh: p=0.85  SBRTtb* - SBRTh: p=0.008 |
| **Gallbladder** | Dmax | 2.01±6.94 | 0.64±7.10 | 0.74±7.12 | I-RT - SBRTtb: p=0.24  I-RT - SBRTh: p=0.28  SBRTtb* - SBRTh: p=0.02 |
| **Esophagus** | Dmax | 0.63±0.33 | 1.94±1.12 | 1.91±1.17 | I-RT* - SBRTtb: p=0.0003  I-RT* - SBRTh: p=0.0001  SBRTtb - SBRTh: p=0.28 |
|  | D1cm³ | 0.51±0.26 | 1.54±1.00 | 1.51±1.03 | I-RT* - SBRTtb: p=0.0009  I-RT* - SBRTh: p=0.0008  SBRTtb - SBRTh: p=0.24 |
| **Kidney** | Dmean | 0.50±0.75 | 0.15±0.99 | 0.17±0.97 | I-RT - SBRTtb*: p=0.001  I-RT - SBRTh*: p=0.01  SBRTtb* - SBRTh: p=0.02 |
|  | D1cm³ | 1.04±3.96 | 0.54±5.39 | 0.59±5.59 | I-RT - SBRTtb: p=0.77  I-RT - SBRTh: p=0.91  SBRTtb* - SBRTh: p=0.003 |
|  | D100cm³ | 0.35±0.48 | 0.08±0.40 | 0.11±0.41 | I-RT - SBRTtb*: p<0.0001  I-RT - SBRTh*: p=0.00003  SBRTtb* - SBRTh: p=0.003 |
| **Heart** | Dmax | 0.94±1.25 | 0.87±2.03 | 1.24±2.10 | I-RT - SBRTtb: p=0.45  I-RT - SBRTh: p=0.23  SBRTtb* - SBRTh: p=0.0001 |
| **Great Vessels** | D1cm³ | 0.56±0.37 | 2.25±0.91 | 2.17±1.05 | I-RT* - SBRTtb: p<0.0001  I-RT* - SBRTh: p<0.0001  SBRTtb - SBRTh: p=0.67 |
| **Rib** | Dmax | 4.36±12.56 | 11.84±7.44 | 11.95±7.39 | I-RT* - SBRTtb: p=0.0002  I-RT* - SBRTh: p=0.0002  SBRTtb - SBRTh: p=0.06 |
|  | D1cm³ | 3.69±6.79 | 10.71±5.99 | 10.84±5.94 | I-RT* - SBRTtb: p<0.0001  I-RT* - SBRTh: p<0.0001  SBRTtb - SBRTh: p=0.07 |
| **Skin** | Dmax | 1.71±1.84 | 6.80±1.83 | 7.30±1.98 | I-RT* - SBRTtb: p<0.0001  I-RT* - SBRTh: p<0.0001  SBRTtb* - SBRTh: p=0.01 |
|  | D10cm³ | 1.21±0.79 | 3.11±0.89 | 3.67±0.82 | I-RT* - SBRTtb: p<0.0001  I-RT* - SBRTh: p<0.0001  SBRTtb* - SBRTh: p<0.0001 |
| **Spinal Cord** | Dmax | 0.47±0.41 | 2.08±1.04 | 2.36±0.96 | I-RT* - SBRTtb: p<0.0001  I-RT* - SBRTh: p<0.0001  SBRTtb - SBRTh: p=0.15 |
|  | D0.35cm³ | 0.46±0.38 | 1.96±0.96 | 2.22±0.88 | I-RT* - SBRTtb: p<0.0001  I-RT* - SBRTh: p<0.0001  SBRTtb - SBRTh: p=0.14 |
|  | D1.2cm³ | 0.44±0.35 | 1.82±0.88 | 2.05±0.80 | I-RT* - SBRTtb: p<0.0001  I-RT* - SBRTh: p<0.0001  SBRTtb - SBRTh: p=0.13 |
|  | D1cm³ | 0.45±0.36 | 1.85±0.89 | 2.08±0.82 | I-RT* - SBRTtb: p<0.0001  I-RT* - SBRTh: p<0.0001  SBRTtb - SBRTh: p=0.13 |
| **Stomach** | Dmax | 0.72±1.70 | 1.82±3.21 | 1.83±2.96 | I-RT* - SBRTtb: p<0.0001  I-RT* - SBRTh: p<0.0001  SBRTtb - SBRTh: p=0.33 |
|  | D10cm³ | 0.48±0.82 | 1.08±1.39 | 1.13±1.27 | I-RT* - SBRTtb: p=0.001  I-RT* - SBRTh: p=0.001  SBRTtb* - SBRTh: p=0.03 |
|  | D1cm³ | 0.63±1.33 | 1.64±2.24 | 1.61±2.11 | I-RT* - SBRTtb: p<0.0001  I-RT* - SBRTh: p<0.0001  SBRTtb - SBRTh: p=0.23 |
